# Everyday experiences of people living with dementia and their carers relating to oral health and dental care

**DOI:** 10.1177/1471301220975942

**Published:** 2020-12-19

**Authors:** Sarah A Curtis, Sasha Scambler, Jill Manthorpe, Kritika Samsi, Yvonne M Rooney, Jennifer E Gallagher

**Affiliations:** Faculty of Dentistry, Oral & Craniofacial Sciences, King’s College London, UK; Faculty of Dentistry, Oral and Craniofacial Sciences, King’s College London, UK; NIHR Policy Research Unit in Health and Social Care Workforce and NIHR Applied Research Collaborative (ARC) South London, The Policy Institute, King’s College London, UK; NIHR Policy Research Unit in Health and Social Care Workforce and NIHR Applied Research Collaborative (ARC) South London, The Policy Institute, King’s College London, UK; Department of Community Special Care Dentistry; King’s Dental Institute, London, UK; Teddington Community Dental Clinic, London, UK; Kingston Community Dental Clinic, Princess Alexandra Wing, Kingston Hospital, London, UK; Global Envoy, King’s College London, UK; Faculty of Dentistry, Oral and Craniofacial Sciences, King’s College London, UK

**Keywords:** dementia, oral health, social care, older people, dentistry

## Abstract

Little is known about how community-dwelling people with dementia, as well as their carers, look after their oral health and use dental care. This exploratory study aimed to explore the beliefs, experiences and practices about oral health of people living with dementia and their carers. We used an ethnographic qualitative approach conducting face-to-face interviews at home with people living with dementia and/or carers. Interview data and field notes were analysed thematically using framework methods. We approached volunteers registered with the online UK. *Join Dementia Research* network from whom a total of 17 participants were recruited in 2018. Five interviews were conducted with carers alone, two with a person living with dementia alone, and five with a carer and person with dementia jointly. Three main themes emerged: *oral health is not prioritised*; *access to dental care is shaped by increasing disability;* and the importance of *continuity of care.* While people living with dementia and their carers may view oral health important once prompted, many reported difficulties in undertaking or assisting with daily self-care and accessing dental services, particularly as dementia progresses. We draw out implications for the organisation and delivery of public and private dental services.

## Introduction

The number of people with dementia is increasing globally and predicted to double every 20 years (Alzheimer’s Disease International, 2019). An estimated 850,000 people are living with dementia in the United Kingdom in 2015 (Alzheimer’s Society, 2017), with numbers predicted to increase to over one million by 2025 and over two million by 2051. Many countries in the global North now have a national dementia strategy, with England’s strategy accompanied by a prime ministerial challenge to improve support (GOV.UK, 2020). Legislation, such as the Care Act 2014 and Mental Capacity Act 2005 (covering England and Wales), provide a framework for dementia care. In addition, the National Institute for Health and Care Excellence (NICE, 2018) has recently outlined what people with dementia and carers should expect from services.

Dementia is characterised by progressive cognitive decline with symptoms that fluctuate in intensity and manifestation (Rockwood et al., 2009). This variability and unpredictability add further challenges to daily, self, assisted and paid personal care. As with other long-term conditions, dementia affects the lives of both the person with dementia and their family, friends and carers (NHS England, 2020) and can impact on oral health and dental care (Bedi, 2015).

In the global North, most people now age with some, or all, of their natural teeth. However, there is evidence of a trend of increased caries (tooth decay) among older people (Bedi, 2015; Newton et al., 2018). As with others, people living with a diagnosis of dementia are susceptible to the full range of oral diseases (e.g., caries and periodontal disease). Daly et al. (2017) found ‘emerging evidence…that people with dementia have poor oral health but the relationship between the two is [unclear]’ (p. 5) and called ‘for primary research into [the] links between oral health and dementia’ (p. 17) (see also Manger et al., 2017; and Fiske et al., 2006). While there is little dental research focusing on people with dementia, the evidence suggests that many do not attend a dental practitioner regularly (Lee et al., 2015). Multiple barriers to accessing dental care have been identified by people who are vulnerable and have other impairments or disabilities (Borreani et al., 2008; British Dental Associtaion, 2013; Chalmers, 2000; Scambler et al., 2015; Vanobbergen et al., 2007). These include environmental, organisational, social and professional barriers, along with a lack of perception of need and the failure to prioritise oral health.

In addition to potential barriers to accessing care, prevention of oral disease largely relies on good daily self or assisted care (Chideka et al., 2015; Public Health England, 2017). With increasing numbers of older people with dementia living at home (Newton et al., 2018), many need support with daily practices of oral health care to prevent diseases and maintain oral health (Daly et al., 2017). Maintaining oral health for people with dementia may involve the person (self-care), carers (family or friends) or paid staff (such as agency employed home care workers and directly employed care workers) as well as the professional dental team.

While there is emerging evidence on the possible association of dementia and oral health (Daly et al., 2017) and oral health challenges (Daly et al., 2017; Delwel et al., 2018; Newton et al., 2018), there is limited literature exploring the day-to-day experiences of dementia and its effects on oral health behaviours and practices – specifically for those living at home. Moreover, research has mostly been conducted in residential facilities (Paley et al., 2009), yet most people experiencing dementia live in the community. This exploratory study sought to address these gaps in support of people living with dementia and their carers.

## Objectives

A multidisciplinary research team including a former carer of a person with dementia was funded by King’s College London to conduct an exploratory study aiming

1. to explore the oral health beliefs, experiences and practices of people living with dementia and their carers;
2. to better understand the impact dementia has, over time, upon oral health and oral care practices, as well as engagement with dental services; and
3. to explore behaviours relating to oral health and health care, including continuity and change.

## Methods

Ethical approval for the study was received (King’s College Research Ethics Committee) in April 2018 (REC Reference Number: HR-17/18–5364). Permission was obtained from the U.K. network *Join Dementia Research* (in which there are >31,000 registered volunteers) to publicise this study on their database to seek potential participants.

Inclusion criteria for participants were those who

1. lived in private or local authority housing (not care homes or supported living facilities);
2. had a diagnosis of dementia or were the adult carer (family or friend) for at least 6 months of someone with dementia; and
3. had capacity to participate in the study and provide informed consent to an interview.

Purposive sampling (Ritchie et al., 2013; Saks and Allsop, 2012) was employed to select participants who met the inclusion criteria and to obtain a diverse range of participants. People with dementia, carers, and the two simultaneously, where appropriate, were approached for interviews. The information sheet and consent form were sent to participants at least 48 hours prior to the interview. Participants were advised that these could be read and completed at their own pace and that seeking assistance was encouraged.

Drawing on a flexible topic guide, informed by the literature, we used a phenomenological approach to collect data (Ritchie et al., 2013). This allowed for in-depth exploration of participants’ experiences and an opportunity to examine oral health practices in everyday life, with a focus on the impact of dementia symptoms – both in terms of self or assisted oral care practices and engagement with dental services. Experiences and views of care and treatment were explored with all participants. By drawing upon social constructions of health and illness as pioneered by Kleinman (1988), specific attention was given to the participants’ experiences, routines, relationships, emotions and techniques, as well as their perceived achievements and challenges in maintaining good oral health.

All interviews were recorded with permission and transcribed verbatim. Thematic analysis was undertaken as informed by the data and the ‘analytic story’^1^ (Corbin & Strauss, 2008). Framework analysis (Gale et al., 2013) was used to organise the data which were all entered into NVIVO, a data software management tool.

## Findings

### Participants

Seventeen people participated in 12 semi-structured interviews. All those who volunteered to participate (bar one)^2^ were recruited for this research. Five interviews were held with a carer alone (P1-5), two with a person living with dementia alone (P1-2) and five jointly with a person living with dementia and their carer (P3-7). Some participants experiencing dementia received support from relatives and neighbours, while others received support from staff such as district/community nurses, care workers, and occupational therapists. Participants’ details are summarised in Table 1.

**Table 1.** Participant demographic characteristics.

Joint/Sole interview	Participant ID	Person with dementia (PWD) Or carer	Age (years) by age band	Gender (M = male; F = female)	Relationship	Living arrangement
Sole	P01	PWD	80–85	M		Lives alone
Sole	P02	PWD	60–65	F		Lives with a son
Sole	P03	Carer	60–65	F	Daughter of mother with dementia	Live apart
Sole	P04	Carer	65–70	M	Son of mother with dementia	Live apart
Sole	P05	Carer	40–45	F	Daughter of mother with dementia	Live together
Sole	P06	Carer	60–65	M	Son of mother with dementia	Live together
Sole	P07	Carer	65–70	F	Wife of husband with dementia	Live together
Joint	P08	PWD	90–95	F	Mother	Live together
	P09	Carer	60–65	F	Daughter	
Joint	P10	PWD	85–90	M	Husband	Live together
	P11	Carer	70–75	F	Wife	
Joint	P12	PWD	60–65	F	Wife	Live together
	P13	Carer	70–75	M	Husband	
Joint	P14	PWD	75–80	M	Husband	Live together
	P15	Carer	65–70	F	Wife	
Joint	P16	PWD	70–75	F	Mother	Live apart

### Themes

#### Oral health is not prioritised

The priority placed on oral health by participants varied, both in its own right and in relation to other competing matters. While participants did think about cleaning teeth and generally reported that they did so; overall, oral health was not considered a primary concern, especially in the context of dementia’s progression and other long-term conditions.

Consistency of oral health practices did not appear to be a priority, amidst many other things to remember, for people with dementia:

*It feels […] incredibly busy [during] the day trying to get it all [health requirements] in. I take lots of tablets and I’ve got to remember… I have my own technique for that… but if I don’t use my technique I don’t even remember [if I’ve taken] my tablets… I’ve got to really concentrate to keep the blood pressure [down]… and so that’s important, so I try and do it [take the tablets] at a set time and that takes precedence over teeth.* (P02. F, person with dementia)

One participant who lives with dementia talked about the difficulties of self-care activities, without prompts from others. She acknowledged forgetting to clean her teeth as well as the important reminders from her son, including visual prompts modelling healthy behaviour:

*My son, he helps …He prompts; he does prompt the medication [verbally]…. He prompts the teeth by doing it himself, so he walks by me with his electric toothbrush.* (P02. F, person with dementia)

Talking about the future, this participant needed, expected and welcomed more prompts, described as ‘happy prompts’ from those helping her. Thus, while teeth and dentures might be brushed, other preventive measures, such as restricting the frequency of sugar intake, were not routinely considered. This became particularly apparent when balanced against other aspects of life and care:

*Yes, the thing is we’ve got to the point now where we’re just grateful that she eats anything. So, I know she doesn’t have the greatest diet, but I just want her to eat I do not care what she eats – as long as it’s something! So, she’s always had a sweet tooth she likes cakes and biscuits, she can’t chew now. [S]he likes meringues and they’re great because they melt you know, I can put a piece in her mouth and it melts and I know it’s full of sugar, but she enjoyed it and I’m satisfied that she ate something and there was a little bit of strawberry you know chopped up in it, so I don’t care. I know it’s awful, but I really don’t care.* (P03. F, carer)

Carers’ accounts suggested that oral health was just one of many routine tasks that had to be performed by the person living with dementia and/or their carers or home care workers. One carer reported that community nurses had not prompted her to maintain her husband’s oral health:

*Yes, and he’s on a modified diet because of his swallow and I do, I mean I’m quite, not obsessive, but I’m very conscious of keeping him hydrated and mixing water in with everything else but on reflection the district nurses don’t mention it [oral health] either, you know they check the catheter, they check the [unclear-0:12:58.3] but they don’t think about the mouth.* (P07. F, carer)

Assisting with oral health did not seem to be routinely included in home care workers’ tasks, although we did not see care plans and did not witness home care workers’ activities. Similarly, dental appointments were often not prioritised or could be overlooked. One participant experiencing dementia reported hoping that he was living in the hope that his teeth would remain functional and pain-free:

*I’m resistant to the idea of going to the dentist. [M]y basic view is I am 87 years old; I’m not going to last probably…I may not last another five or 10 years and if my teeth can last that time, there’s no point in worrying about them before then. That’s my view.* (P10. M, person with dementia)

Furthermore, the importance of making dental care appointments seemed to be undermined by barriers encountered in accessing dental services. This included problems with physical access, meeting the cost of treatment, or finding local dental services that were willing to make home visits for check-ups and not just emergencies. This reflects the main finding in this theme which was that oral health was only prioritised when it obviously impacted on comfort or physical or social functioning:

Interviewer*: And do you think you have good or bad oral health?*

Participant: *I think I have good oral health.*

Interviewer*: And what makes you say that?*

Participant*: I don’t suffer any pain.*

…

Interviewer*: How would you know if you’ve got problems?*

Participant*: Oh, I’d feel it.*

…

Interviewer*: Do you currently have any pain?*

[The answer was no]

…

Interviewer: *If you did you would go to the dentist if it was on-going?*

Participant*: I did have some pain, and I just waited to see if it went away or not, and it did so I… If it had persisted, I would have, the idea had crossed my mind but then it disappeared and I said okay, I*'*ll postpone that.*

(P10. M, person with dementia)

Other participants prioritised dental care when facial aesthetics were involved, rather than pain. One person referred to a problem with a crown on her tooth which had recently been successfully restored following several instances of it falling off. She had not experienced pain but was embarrassed about the gap in her teeth and wanted to visit her dentist as soon as possible to have it fixed:

*I was desperate [to see the dentist], I can’t go anywhere…I won’t speak to anyone [until it is fixed].*

(P12. F, person with dementia)

Prompted by engagement with this research, some participants suggested that oral health had already become a greater concern and priority.

#### Access is shaped by increasing disability

Behaviours, such as attending a dental practice, which had been previously routine, reportedly changed as dementia progressed. Access to oral health care and services appeared to be shaped by increasing disability as dementia progressed. With the cognitive and physical decline that dementia brings, behaviours may change and levels of disability rise, impacting on both the person living with dementia and their carer. This was illustrated by one participant who described his wife’s growing symptoms of severe anxiety, rendering it impossible for her to visit the dentist by herself. This exposed her reliance on him to help her access any services outside the home:

*[She has] got anxiety so she can’t go anywhere on her own. There’s no way she could go on [to the dentist] her own.* (P13. M, carer)

Increasing reliance on others was evident. Participants living with dementia and carers highlighted the need to receive support with the general activities of everyday life during the dementia journey. Carers reported navigating a complex health and care system and multiple service providers who often did not seem to communicate efficiently with one another. In this context, accessing dental services emerged as a particular problem; it was not said to be routinely included in ‘packages of social care’, and there appeared to be little information available on how to access dental services when needed:

*I haven’t had any contact with the NHS dental service with mum at all and then last year she broke a tooth and I didn’t know where to go or who to contact. I got ferried around from pillar to post, forms to fill in, different people to call and it was all very hard, and it should’ve been simple and then I found out there was an incredibly long waiting list... So, it seems like the one, solitary mobile dentist who’s doing the whole of the (area) [has] got a huge waiting list, in the meantime she’s got a broken tooth … I was quite shocked when that wasn’t deemed an emergency. [It was] rubbing her gum but that wasn’t considered enough of an emergency…* (P03. F, Carer)

Difficulty accessing NHS dental care in this situation had led to an extreme family solution:

*…So thankfully, it sounds terrible, but her brother had a file and he filed it down…and that took off the edge but, in the meantime, I thought “what am I going to do”, and I was panicking. So, I started ringing people you know, off the internet, private dentists and they were just astronomically expensive, I mean I would’ve paid it, but it seems as if they were geared to work towards nursing homes anyway and [not] to come out to someone individually*. (P03. F, Carer)

Pain and discomfort, the potential impaired ability to eat and the difficulty finding dental care or long waiting times were all considered to be particularly problematic.

As with all other aspects of life with dementia, increasing disability impacted on both the person living with dementia and their carer. This affected the ability of both to access professionals of many kinds, including dental care. One carer spoke about his difficulty in attending his own regular dental appointments as a result of needing to support his wife who also had anxiety as a complication from her dementia. He was unable to leave her alone and so had to take her to his appointments:

*There is a factor that I go to the dentist less now because I’m taking [his wife] all the time and I can’t go on my own, so if anybody’s affected it’s actually me… I can’t go on my own because I need to take you [his wife] and it’s just not easy.* (P13. M, carer)

This was echoed by another carer who had attended regular dental check-ups but was now unable to do so due to the time constraints associated with caring for her husband and his restricted physical mobility:

*I don’t have time to go for lots and lots of treatment and sort things out…We’re waiting for a wheelchair, the physio yesterday tried to hurry it up, but we’ve been told it’s going to be another 2 months…We’d only be able to get physically there in a taxi, [and] we would need to do it on a day that our daughter was around to help get him down the stairs*. (P15. F, carer)

Difficulties with physical access were location- and provider-specific, and some participants reported that their local dental practices were inaccessible. Challenges in accessing dental services increased when general dental practitioners and community services could no longer be accessed directly, and domiciliary dental care was required. Carers adopted various strategies to seek and maintain such care. One carer recounted the difficulties in arranging a dental hygienist to visit her ‘bedbound’ mother at home. On finding that the visit was a ‘one off’, she ‘smooth-talked’ the hygienist to visit again.

*[I]t sounds terrible but I’m going to schmooze her a bit because that’s what I did the first time. When she said this is it [no more visits would occur], I said ‘you know mum really enjoyed this and I’ve learnt a lot’ and I think because you know, I talk to anybody and I think I’m quite good with people and she said ‘oh I think we could do one more visit’ but then she explained the discharge and blah, blah, blah. So, I’m going to try and schmooze her again on Friday to see if she’ll come out one more time and one more time, but I shouldn’t have to do that, she may be in a bad mood on Friday and say no that’s it.* (P03. F, carer)

There seems to be a lack of information for people with dementia and their carers about what dental care is available and can be expected locally. Participants in this study felt that local services were overstretched, and there was a perception that dentists were only able and willing to respond to acute emergencies, rather than providing services that carers believed to be in their relative’s best interests. This raises questions about expectations of care and potential mismatches between lay and professional views on good care for people with dementia.

In summary, gaining access to dental services was increasingly important, and yet more difficult, as dementia progressed, affecting both the person living with dementia and their carers. Mobility problems, financial constraints, accessing different agencies and decreasing independence affected the daily life, including oral health, of both people living with dementia and their carers. One factor that seemed to help was continuity of care.

#### Continuity of care

Challenges in relation to receiving continuity of care pertained to dental practitioners and wider dental team members, as well as among those involved in home care provision, with a particular focus on communication. There was evidence of the importance of the relationship of people experiencing dementia with their dental practitioners as outlined below:

*Oh, it makes a huge difference…. Yeah, it really makes a big difference because as you said I do have anxiety but because I have been going to see him [the dentist] so often I got to know him very well and he’s always very respectful, he always tells you when you go in, he’ll say, oh what have you been doing, breaking [past] the fear… he makes you very comfortable and the continuity is important*. (P12. F, person with dementia)

While continuity of personnel can promote trust, it was not always possible to access the desired clinician. Connection with the past was also important. One carer recounted how her mother had always been extremely conscious of her teeth and smile and wanted to see a dentist from her own ethnic background; however, the carer was unable to navigate the system to find such a person:

*[I]n regard to the teeth it was quite stressful. She kept saying she wanted to go and see a [specific] dentist. She knew she was comfortable with them [dentists from her cultural background] and trusted them and they always made her really relaxed…she was like when am I going to go. So, every day, at least for 6 months [she was asking] when am I going to the dentist, take me to the dentist, I want to see my dentist.* (P05. F, carer)

Continuity of care cannot be guaranteed when delivering formal services in the home. Here, family carers felt that information sharing became important, particularly around routine personal care activities such as tooth brushing and denture care. Without care provided by the same people, or good communication between staff and family, it seemed very difficult for all carers to know what service provision had occurred or was required:

*Well I read [the care workers’] log every day and sometimes they put you know the morning wash … clean teeth, I don’t know if, I’m presuming they do it every day because sometimes they don’t write everything down, because it’s so run of the mill they just don’t do it every day, some of them write it some of them don’t. So, I am presuming that they are doing it although I do it as well, so she may be getting her teeth cleaned twice a day I don’t know.* (P03. F, carer)

Without continuity and the trust that it engendered, the ability to maintain self and assisted oral health care appeared to be further jeopardised.

## Discussion

The findings of this exploratory study suggest that for people with dementia, oral health does not emerge as a priority until problems arise. Participants suggest that in England, current oral health care can be insufficient for some people with dementia, both in relation to daily self or assisted care within the home and access to professional dental services, particularly as their condition progresses.

Effective self-care involving a healthy diet and good oral hygiene with a fluoride toothpaste forms the basis of routine daily self-care, supported by contemporary evidence (Public Health England, 2017). However, as dementia progresses, people are increasingly reliant on others for assistance (Rees et al., in press), meaning self-care becomes more complex. Dietary changes may, of course, be associated with the experience of dementia, rather than oral disease (Ikeda et al., 2002), yet we need to further explore and understand the advice of health professionals from different disciplines and people experiencing dementia from differing cultural and socioeconomic backgrounds in order to determine the likely causes and problems of poor oral health overall. Equally, self-perceived oral health needs and values vary in seriously ill older people and their families as explored by Chen et al. (2015). This research and the wider literature therefore suggest that care services should respect the different values of individuals when seeking to best address the oral health needs of seriously ill people and their family – particularly for individuals who value oral health and related quality of life in their entire life (Chen et al., 2015).

Regular dental care is important to maintain oral health (NICE, 2004), particularly into older age (Gallagher, 2019). Adults are recommended to attend a dentist at least once every 24 months, with people at higher risk of disease, such as vulnerable older people, advised to attend more frequently, based on their level of risk (NICE, 2004). As noted above, dementia is a cognitive disability; and thus, there is an equality dimension to the adequacy of access to oral health services (as legislated for in the UK Equality Act 2010).

Our exploratory study suggests that time, knowledge, skills and clear pathways are needed to understand how to access and navigate services and which service deficits need quantifying and addressing. We do not know if people are routinely told about dental services in postdiagnostic discussions at memory clinics or in the mandatory primary care reviews; this needs further exploration. Dementia-specific health and social care providers should have information on local, accessible dental services and how to access them and ideally actively support their clients in doing so. Dental practitioners could also helpfully make a stronger case when treating those experiencing dementia and their carers, and optimal dental care is important to support their wider quality of life – including preventing difficulties in eating, pain and discomfort, communication and aesthetics (Kandelman et al., 2008). Also, recognising the pressures on family carers themselves during this phase of life (Brimblecombe et al., 2018) is part of the professional task.

Oral health priorities differed, regardless of the dementia or caring experience. The accounts presented here suggest that it is often the physical and social impacts of poor oral health that prompt contacts with dental services, rather than planned attendance. However, both good self-care and regular access to dental services to manage disease early and maintain prevention seem to become problematic and more important as dementia progresses. When overall oral health is not considered a priority, access to services and service provision for vulnerable groups becomes reactive, instead of preventive, putting the person living with dementia at greater risk of overall poor health and diminished quality of life. Our exploratory study suggests that continuity of care may be particularly important for people living with dementia (Cornwell, 2012; Larsen et al., 2019; Sonola, 2012) and may be more so for those who are dentally anxious. This concept of continuity of care is supported by the wider literature, which also suggests that living with dementia impacts mortality and increases early or unwanted moves to care facilities as well as negative outcomes for people with multiple, long-term conditions. (Amjad et al., 2016; Snowden et al., 2017).

Chen et al. (2015) highlight that self-perceived oral health needs and values vary in seriously ill older adults and their families. This research and the wider literature suggest that care delivered should respect the particular needs of this community to best address providing good oral health to those experiencing dementia, and to respect the value placed on oral health and related quality-of-life features (Chen et al., 2015). That said, the Faculty of General Dental Practice (2017a) in the United Kingdom do have a comprehensive set of guidelines on dementia-friendly dentistry. Our findings however raise important questions about NHS dental systems in which dental team members have less flexibility to deliver care to vulnerable groups who may require more time for care to be delivered in different settings (GOV.UK, 2019). Their care and treatment may need to be scheduled for their ‘better days’. Also, liaison with carers and sensitive management of the process key to good outcomes (Faculty of General Dental Practice, 2017b) – all of which require time. This is complex, especially when front-line dentists themselves are displaying signs of dissatisfaction with the NHS system (NHS Digital, 2018; Salazar et al., 2019).

This exploratory research is timely, given the emphasis on integration of care, highlighted in the recent NHS Plan (NHS England, 2019), with implications for integration of dental care across future health systems, in support of people living with dementia. The NHS England commissioning guide for Special Care Dentistry highlights population growth and the importance of Interdependency of the Special Care Dentistry Managed Clinical Network, “with clinical networks including those for long term conditions and dementia”, but does not go further than this (NHS England, 2015). These services seemed to be invisible to participants in our study. Better understanding of oral health practices and dental services among frontline dementia practitioners may help promote oral health, with oral health being included in local dementia action plans and being taken up by dementia champions. Any revisions to the National Dementia Strategy and the Carers’ Action Plan (Department of Health and Social Care 2018) could set quality indicators for oral health care and dental services. Furthermore, we need to ensure that skills, knowledge and awareness are addressed in the training and continuing professional development of all practitioners to enable and maintain self and assisted oral health care. Further research and action are required to explore how care can best be delivered for this group to support our ageing population (Gallagher, 2019).

The limitations of this exploratory study are acknowledged. The research fieldwork was conducted in one area of England (South East) and therefore may not be nationally representative. Participants are more likely to have been from social groups that place value on research generally and have the time and interest to contribute. These are also likely to be people who are better at negotiating health and social care systems. While it could be argued that the sample size is small, it is in line with other qualitative explorative studies of this type (Bissett et al., 2013; Dharamsi et al., 2010; O’Reilly & Parker, 2013; Tinker et al., 2018 see also). Furthermore, as an initial exploratory study, it was not able to explore people’s views through the stages of dementia – this being an area that requires further research to better inform future care. Nonetheless, nuanced and rich data were realised from the interviews, and saturation was reached in that no new themes emerged in later interviews of this cohort which highlights the importance of longer term research.

## Conclusion

This exploratory study highlights some of the challenges faced by people with dementia and their carers and the limitations of support for their maintenance of oral health. It appears that oral health often only becomes a priority when problems arise. In comparison to general overall health, oral health seems to be less visible. Our findings have raised questions about access in routine and in emergency situations, consistency of access and the delivery and promotion of good oral health care for people with dementia and their carers.

## Notes

1 The description of “experiences, spoken words, actions, interactions, problems, and issues expressed by participants” (Ritchie et al., 2013, p.51).

2 One person indicated interest to participate by telephone yet was unable to be contacted by the researcher after several attempts.
